# Fatty Acid Composition and Fatty Acid Associated Gene-Expression in Gilthead Sea Bream (*Sparus aurata*) are Affected by Low-Fish Oil Diets, Dietary Resveratrol, and Holding Temperature

**DOI:** 10.3390/md16100379

**Published:** 2018-10-10

**Authors:** Claudia Torno, Stefanie Staats, Stéphanie Céline Michl, Sonia de Pascual-Teresa, Marisol Izquierdo, Gerald Rimbach, Carsten Schulz

**Affiliations:** 1 GMA–Gesellschaft für Marine Aquakultur mbH, Hafentörn 3, 25761 Büsum, Germany; michl@gma-buesum.de (S.C.M.); cschulz@tierzucht.uni-kiel.de (C.S.); 2 Institute of Animal Breeding and Husbandry, University of Kiel, Olshausenstraße 40, 24098 Kiel, Germany; 3 Institute of Human Nutrition and Food Science, University of Kiel, Hermann Rodewald Straße 6, 24118 Kiel, Germany; staats@foodsci.uni-kiel.de (S.S.); rimbach@foodsci.uni-kiel.de (G.R.); 4 Department of Metabolism and Nutrition, Institute of Food Science, Food Technology and Nutrition (ICTAN–CSIC), José Antonio Novais 10, 28040 Madrid, Spain; soniapt@ictan.csic.es; 5 Grupo de Investigación en Acuicultura (GIA), Instituto Universitario Ecoaqua, Universidad de Las Palmas de Gran Canaria, Crta. Taliarte s/n, 35214 Telde, Las Palmas, Canary Islands, Spain; marisol.izquierdo@ulpgc.es

**Keywords:** stilbene, EPA, DHA, ∆6-desaturase, bioactive, PPARα, omega-3 fatty acid

## Abstract

To sustainably produce marine fish with a high lipid quality rich in omega-3 fatty acids, alternative sources of eicosapentaenoic acid (EPA) and docosahexaenoic acid (DHA) are being identified. Moreover, the use of bioactive compounds that would stimulate the in vivo fatty acid synthesis, such as resveratrol (RV), would reduce the dependence on fish oil in aquafeeds. Gilthead sea bream (*Sparus aurata*) were fed four experimental diets combining two fish oil levels (6% dry matter (DM); 2% DM) with or without 0.15% DM resveratrol supplementation (F6, F2, F6 + RV, F2 + RV) for two months. Additionally, the fish were challenged either at 19 °C or 23 °C. A higher water temperature promoted their feed intake and growth, resulting in an increased crude lipid content irrespective of dietary treatment. The fatty acid composition of different tissues was significantly affected by the holding temperature and dietary fish oil level. The dietary RV significantly affected the hepatic EPA and DHA content of fish held at 19 °C. The observed effect of RV may be partly explained by alterations of the mRNA steady-state levels of ∆6-desaturase and β-oxidation-related genes. Besides the relevant results concerning RV-mediated regulation of fatty acid synthesis in marine fish, further studies need to be conducted to clarify the potential value of RV to enhance fillet lipid quality.

## 1. Introduction

Fish are the predominant source of the omega-3 (n-3) long-chain polyunsaturated fatty acids (LC-PUFAs), eicosapentaenoic acid (EPA, 20:5n-3) and docosahexaenoic acid (DHA, 22:6n-3) which play a unique role in human nutrition, health, and development [1]. To produce fish rich in EPA and DHA, aquafeeds contain fish meal and fish oil obtained from wild catches. Current trends demanding a more sustainable and economic fish production have led to an increased development and inclusion of alternative terrestrial and plant ingredients in aquafeeds. Depending on the alternative ingredients used, the health and nutritious properties of the farmed fish may be affected [2]. The replacement of dietary fish oil by vegetable oils might be feasible in some species, but can be problematic especially in carnivorous marine species [2]. The gilthead sea bream (*Sparus aurata*) is a carnivorous marine fish species of economic importance especially in the Mediterranean region [3]. Sustainable diet formulations with low levels of fish oil do not necessarily affect the growth and performance, but impair the fillet quality and fatty acid (FA) composition of sea bream and European sea bass (*Dicentrarchus labrax*) [4,5,6,7]. The FA composition of marine species usually reflects that of their diet, since the ability to convert dietary C18 precursor fatty acids to LC-PUFAs is hardly present in marine finfish [8,9]. Nowadays, farmed gilthead sea bream can have decreased EPA and DHA contents per gram of fillet in comparison to past years due to fish oil replacement [5], although the content in farmed sea bream is higher than in the wild specimens [4].

To counteract this reduction trend, recovering the EPA and DHA levels, and to wash out undesirable FA of vegetable origin, finishing diets high in fish oil have been successfully used in sea bream [4,6]. Furthermore, the use of microalgae containing LC-PUFA in the diets of marine finfish, such as *Pavlova viridis* in European sea bass diets [10], is also a promising approach. The use of *de novo* n-3 oils from genetically modified oil crops, for example, in Atlantic salmon (*Salmo salar*) and sea bream [11,12], is innovative, but due to legislation, it is not practicable in all countries.

The aforementioned approaches have one thing in common: They rely on already existing sources of EPA and DHA that are supplied to the fish via the diet. Thus, any improvement of the innate ability of the fish to cope with diets low in EPA and DHA would be an interesting alternative. The modification of the underlying molecular mechanism and exploitation of genetic capacities build the backbone for this approach. In freshwater fish that have a limited natural ability to convert the precursor C18 FA α-linolenic acid (ALA, 18:3n-3) to EPA and DHA [13,14], bioactive phytochemicals might stimulate this FA synthesis. An elevation of the EPA and DHA levels has successfully been shown in rainbow trout (*Oncorhynchus mykiss*) fed diets containing resveratrol [15] and sesamin [16], in zebrafish (*Danio rerio*) embryos exposed to wine polyphenols [17], and salmon hepatocytes treated with genistein [18] and sesamin [19]. Apart from the hardly present in vivo FA bioconversion in marine fish, some species like sea bream and sea bass seem to possess the genetic capacity to perform the synthesis at least partly [20,21,22,23,24]. Thus, it might be possible to exploit the genetic capacity of marine finfish and activate the expression of dormant genes encoding proteins involved in the FA synthesis. Bioactive secondary plant compounds that increased the endogenous FA synthesis of freshwater fish might be a promising tool to be investigated in marine finfish.

Resveratrol (RV) is a stilbene derivate produced by plants, mainly grape vines, in response to infections [25] and has potential health-beneficial, anti-inflammatory, and anti-oxidant properties [26,27,28]. The possible modulation of animal lipid metabolism [17,29,30] and the potential to increases elongase and desaturase (∆6- and ∆5-desaturase) activities [31] are interesting for its application in fish. The fact that RV indicated the increasing properties of LC-PUFA in mammalian cell cultures [31,32], zebrafish [17], and rainbow trout [15], makes it an interesting phytochemical for nutrition studies in marine fish.

Additionally, environmental factors may influence the content of n-3 LC-PUFA or the expression and activity of enzymes involved in the FA synthesis in fish [33,34,35]. In freshwater fish and salmonids, it seems that the FA desaturation, elongation, and β-oxidation activities are increased at lower temperatures [33,34]. Studies with marine fish reveal controversial results. Vagner et al. [36] demonstrated that temperature did not affect the diet-induced upregulation of the ∆6-desaturase (∆6-D) in sea bream larvae. In contrast to that, Skalli et al. [35] demonstrated that the amount of LC-PUFAs increased in the phospholipid fraction in sea bass held at low water temperatures of 22 °C. Since temperature affects diet intake and growth, it seems likely that the FA synthesis might eventually be affected. Thus, an investigation of phytochemical-induced effects in sea bream under the influence of nutritional and environmental factors is highly interesting.

The aim of this study was to investigate the effects of low-fish oil diets supplemented with dietary RV on (1) growth, (2) performance parameters, (3) whole body nutrient composition, (4) FA composition of the whole body, liver and fillet, and (5) the mRNA steady-state levels of selected genes encoding proteins involved in the desaturation (∆6-D), FA metabolism (Carboxyl ester lipase (CEL)), and β-oxidation of FAs (Peroxisome proliferator-activated receptor α (PPARα); Enoyl-CoA hydratase (ECH)) of the gilthead sea bream. Furthermore, we addressed the question whether the holding temperature played a role in the response of the investigated parameters.

## 2. Results

### 2.1. Growth and Performance Were Affected by Temperature but Not by Dietary Treatment

During the experimental period of eight weeks, all experimental groups exhibited overall good growth and performance (Table 1). The sea bream held at 19 °C doubled their weight (approx. 2.2 fold) and fish held at 23 °C tripled their weight (approx. 3.4 fold). The specific growth rate (SGR) of fish held at 19 °C was between 1.3% d^−1^ (F2 + RV) and 1.6% d^−1^ (F6). Fish held at 23 °C had higher SGR with values between 2.2% d^−1^ (F2 + RV) and 2.4% d^−1^ (F6 + RV), respectively. The same applied for the daily feed intake (DFI) which was about 3.2 for fish held at 19 °C and 4.2 for fish held at 23 °C. Fish of each feeding treatment had a significantly lower final body weight (FBW), SGR, and DFI at 19 °C in comparison to 23 °C (*p* < 0.05, Table 1, indicated by *). The feed conversion ratio (FCR) remained unaffected by the dietary treatment or temperature challenge and ranged from lower values of 1.9 (F2 + RV at 23 °C) to higher values of 2.5 (F2 + RV at 19 °C). The protein efficiency ratio (PER) and protein productive value (PPV) differed only in the tendency between the same dietary treatments held at different temperatures (F2 and F2 + RV). The Hepatosomatic index (HSI) differed within the same dietary treatment between the two holding temperatures and reached higher values in fish held at 19 °C. When fish were fed the RV-supplemented diets and held at 19 °C, a significantly elevated HSI was present in the group F2 + RV in comparison to the group F6 + RV (*p* < 0.05, Table 1, indicated by m, n). The Fulton condition factor (FCF) did not differ between the dietary treatments, but between the two holding temperatures with significantly higher values at 23 °C. The evaluation of the statistical significance of effects proved that all growth and performance parameters were affected by the holding temperature in the first place (Table 2).

### 2.2. Whole Body Nutrient Composition Was Predominantly Affected by Holding Temperature and Secondly by Dietary Treatment

After the eight week experimental period, the whole body nutrient composition of fish differed based on the two holding temperatures, except for crude protein (Table 1 and Table 2). The fish had significantly higher dry matter (DM), crude lipid, and gross energy and significantly lower crude ash when held at 23 °C (*p* < 0.05, Table 1, indicated by *). Additionally, fish held at 23 °C and fed the control diet with 6% DM fish oil (F6) had significantly elevated crude lipid in comparison to fish fed the fish oil-reduced diet F2 (*p* < 0.05, Table 1, indicated by a, b). A similar trend was observed at the lower holding temperature. The RV-supplementation tended to decrease crude lipid and gross energy in fish fed the diet F6 + RV in comparison to fish fed diet F6, both held at 19 °C (*p* < 0.1, Table 1, indicated by (A), (B)). The evaluation of the statistical significance of effects indicated that crude lipid was predominantly affected by the interaction of dietary fish oil and RV-supplementation (Table 2). Similar tendencies (*p* < 0.1) were visible for gross energy and DM.

### 2.3. Fatty Acid Composition of Different Tissues of Sea Bream was Predominantly Affected by the Holding Temperature and the Dietary Fish Oil Level

#### 2.3.1. Whole Body Homogenate

The overall FA composition of the sea bream’s whole body homogenates did not fully reflect the FA composition of the diets (Table 3), but instead, was predominantly affected by the interaction of the holding temperature and the dietary fish oil level (Table 2). Based on the different holding temperatures, the amounts of saturated FAs (SFAs) and monounsaturated FAs (MUFAs) were significantly higher in fish held at 23 °C, whereas the amount of PUFAs was significantly lower in those specimens (*p* < 0.05, Table 4, indicated by *). Furthermore, the level of MUFA was significantly elevated in fish fed the F2 + RV diet in comparison to fish fed the F6 + RV diet for both holding temperatures (*p* < 0.05, Table 4, indicated by m, n). The same applied for feeding groups F2 and F6, held at 23 °C. The reversed pattern was visible with regard to EPA + DHA and the n-3/n-6 ration, with few exceptions. Dietary RV had almost no effect on the FA composition of the whole body homogenate with one exception. The amount of MUFA was significantly reduced in fish held at 19 °C and fed with the F6 + RV diet in comparison to the F6 diet (*p* < 0.05, Table 4, indicated by A, B), but not in fish fed the F2-based diets. This was also indicated by the evaluation of the statistical significance of effects, which showed that not only an interaction of dietary fish oil level and RV, but also an interaction of dietary fish oil level and holding temperature affected the MUFA content (Table 2).

#### 2.3.2. Liver Tissue

The SFA, MUFA, and PUFA content and EPA/DHA ratio of liver tissue were only slightly affected by the temperature and dietary treatment (Table 5). Overall, the FA composition of the liver tissue seemed to be rather affected by the dietary treatment (fish oil and RV) than the holding temperature (Table 2). All fish fed the F6 and F6 + RV diets had significantly higher n-3/n-6 ratios compared to the corresponding F2 and F2 + RV feeding groups, respectively (*p* < 0.05, Table 5, indicated by a, b and m, n, respectively). The amount of EPA + DHA was significantly affected by the interaction of all three factors, namely temperature, dietary fish oil, and RV-supplementation (Table 2). Significantly higher EPA + DHA values were present in the livers of fish held at 19 °C in comparison to the specimens held at 23 °C (*p* < 0.05, Table 5, indicated by *). Additionally, fish at both holding temperatures fed with the F6 + RV diet had significantly higher EPA + DHA values in comparison to fish fed the F2 + RV diet (*p* < 0.05, Table 5, indicated by m, n). Dietary RV significantly affected the fish held at 19 °C, but not at 23 °C. Fish fed the F6 + RV diet had significantly higher amount of EPA + DHA in comparison to fish fed the F6 diet (*p* < 0.05, Table 5, indicated by A, B), whereas the opposite applied for fish fed the F2-based diets (*p* < 0.05, Table 5, indicated by M, N).

#### 2.3.3. Fillet Tissue

In the fillet tissue of sea bream, the FA composition was affected by the interaction of dietary fish oil and temperature, except for MUFAs, which were only affected by the dietary fish oil level (Table 2). Overall, the amount of SFA was significantly increased in fish held at 23 °C in comparison to fish held at 19 °C (*p* < 0.05, Table 6, indicated by *). The opposite applied for PUFA (all diets) and for EPA + DHA and n-3/n-6 (only for F2-based diets). The feeding of the F2-based diets led to reduced amounts of SFA and reduced n-3/n-6 and increased amounts of MUFA. At both temperatures, the amount of EPA + DHA was significantly higher in fish fed the F6 + RV diet in comparison to the F2 + RV diet (*p* < 0.05, Table 6, indicated by m, n). The same applied for fish fed the F6 diet in comparison to the F2 diet, but only at the 23 °C holding temperature (*p* < 0.05, Table 6, indicated by a, b).

### 2.4. The Gene Expression in the Liver Was Predominantly Affected by Dietary Fish Oil Level

**Figure 1:**
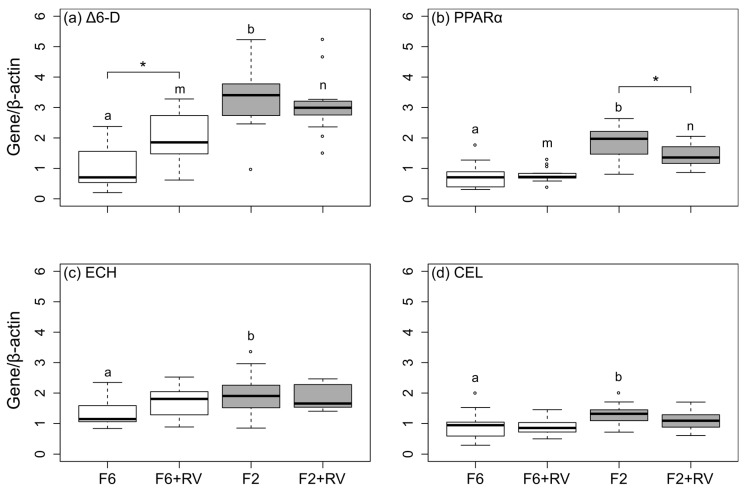
The hepatic mRNA steady-state levels in livers of gilthead sea bream held at 19 °C and fed different diets over a duration of eight weeks. The fish were fed with the F6 and F2 basal diets (6% DM and 2% DM fish oil, respectively) and the experimental diets supplemented with 0.15% DM resveratrol (+RV diets). (**a**) ∆6-D: ∆6-desaturase, (**b**) PPARα: peroxisome proliferator-activated receptor α, (**c**) ECH: enoyl-CoA hydratase, and (**d**) CEL: carboxyl ester lipase were measured in the liver of fish using quantitative real-time RT-PCR and were normalized to the housekeeping gene β-actin. Boxes represent values (n = 15) between the 25 and 75 percentiles, whiskers indicate 1.5 SD; the solid line indicates the median; circles represent values above and below SD. Significant differences (*p* < 0.05) were analyzed using multiple contrast tests for relative effects. Tests were based on the comparisons of dietary fish oil level within one supplement group (no supplement: a, b; + RV: m, n) or supplement type within one dietary fish oil level (indicated by *).

The mRNA steady-state levels of selected genes were only measured in livers of sea bream held at a lower temperature (19 °C) (Table 4). The mRNA levels of ∆6-D, PPARα, ECH, and CEL were affected by the dietary treatment (Figure 1). The reduction of the dietary fish oil level from 6% DM (F6-based diets) to 2% DM (F2-based diets) significantly elevated the mRNA steady-state levels of all four genes (*p* < 0.05, Figure 1, indicated by a, b and m, n, respectively). The fish fed the F6 + RV diet had significantly elevated hepatic mRNA steady-state levels of ∆6-D in comparison to fish fed the F6 basal diet (*p* < 0.05, Figure 1a, indicated by *). Furthermore, the mRNA level of PPARα was significantly reduced in the livers of fish fed the diet F2 + RV in comparison to F2 (*p* < 0.05, Figure 1b, indicated by *).

## 3. Discussion

### 3.1. Growth, Performance and Nutrient Utilization

Throughout the experimental period of eight weeks, the juvenile sea bream of all groups exhibited good growth and performance. The diet formulation with a crude lipid content of only 14.5% DM and strictly reduced fish meal (5% DM) and fish oil (2% DM) content did not impair the fish growth and performance. The ability of sea bream to cope with diet formulations reduced in marine ingredients has previously been shown, but extruded diets with a higher crude lipid content (approx. 25% DM) and higher fish meal and fish oil inclusion rates had been used [4,6,8,9,37]. The combined replacement of fish meal and fish oil (15% DM and 5% DM, respectively [38] and 12.5% DM and below 4.4% DM, respectively [39]) with plant ingredients had previously led to a growth depression of gilthead sea bream. It is commonly accepted that sea bream need minimal amounts of n-3 LC-PUFAs and a minimum EPA + DHA amount of 0.9% of a dry diet for good growth and performance [40,41]. It is noteworthy that the sea bream in this trial exhibited no growth and performance depression with diets reduced in fish oil, although the amount of EPA and DHA was only 0.37% for the dry diet and thus below the generally accepted recommendations.

The distinctive impairment of DFI, SGR, FBW, and FCF at 19 °C represents the normal adaptions of the sea bream to lower temperatures and is in accordance with previous findings [42,43]. The SGR (1.3–2.4% d^−1^) was within the range or even better than those previously reported by other studies with sea bream of comparable size held at similar water temperatures (1.2–1.6% d^−1^ [44] and 1.8–1.9% d^−1^ [8]). The DFI (3.2–4.3% d^−1^) and FCR (1.9–2.3) were higher than reported in the aforementioned studies. Additionally, the nutrient utilization parameters PER (approx. 1.1) and PPV (approx. 16.4%) were at the lower margin as previously reported (PER: 1.02–1.51 [38,44] and PPV: 17.7–23.6% [44]). The diets low in fish meal (5% DM) and high in plant ingredients might have affected the nutrient utilization and thus the PER, PPV, and FCR. Furthermore, the DFI was calculated based on the weighed daily feed ratios and the fact that some feed was lost due to the water current cannot be excluded. Thus, these values might overestimate the actual DFI and, in return, might result in an underestimation of the actual FCR, PER, and PPV. The effect of reduced dietary fish oil content on the HSI of sea bream was most pronounced in fish held at the lower water temperature and fed RV-supplemented diets. Generally, the HSI of sea bream increases when the amount of dietary vegetable oil is increased [9,38], which was only partly visible within this study.

### 3.2. Whole Body Nutrient Composition

The whole body crude lipid content was increased in sea bream held at the higher water temperature, in agreement with the slight but non-significant increase previously observed in sea bass [35,45]. In the present study, the increased DFI of groups held at 23 °C might have contributed to this effect. Furthermore, a reduction in the whole body crude lipid conditioned by reduced dietary fish oil inclusion as observed in this study has been previously observed in sea bream [40] and might be a result of the qualitative differences in the used dietary oil sources. The dietary supplementation of RV to the control diet containing 6% DM fish oil led to a technically reduced whole body crude lipid level and gross energy level. A similar statistical tendency towards a reduced whole body crude lipid level had been observed in studies from our group with rainbow trout fed RV-supplemented diets (Torno et al. accepted). The possible explanations might be a reduced lipid digestibility as observed in rainbow trout [46], or metabolic changes (modulation of adenosine monophosphate-activated protein kinase and the sirtuin pathway), amongst others, as extensively investigated in obesity and body weight management studies [26].

### 3.3. Fatty Acid Composition of the Whole Body, Liver, and Fillet

Irrespective of the dietary treatment and temperature challenge, all sea bream had visibly increased amounts of MUFAs in their tissues compared to the diets. The amount of MUFAs can be mainly attributed to the FA 18:1n-9. Furthermore, it seemed that the sea bream readily took up all EPA and DHA offered via the diet, whereas less of the FAs 18:3n-3 (ALA) and 18:2n-6 were taken up. Previous studies reported, that the FA composition of sea bream tissue greatly reflects the dietary FA composition [8,9,39]. The high MUFA content of the sea bream from this trial might be mediated by the predominantly vegetable diet formulation. An increase in MUFAs is often associated with an increase in dietary plant oils and raw materials rich in carbohydrates [4,8,39], supporting the increase of MUFAs in sea bream fed the fish oil-reduced diet in comparison to the control diet in this study. Furthermore, a higher MUFA to SFA ratio was present in the fish held at 19 °C and might be an adaptation to the lower temperature in order to maintain physiological and cellular functions [47,48].

As it could be expected, PUFA and EPA + DHA content in the whole body and fillet was decreased by the reduction in dietary fish oil level, particularly when fish were held at the higher water temperature. If we assume that the change in the tissue FA profile follows a simple dilution model (dilution of tissue FAs by dietary FAs), the fast growth and high tissue FA turnover of fish held at 23 °C would accelerate the dilution process [6]. Furthermore, Izquierdo et al. [4] demonstrated that the greatest change in the FA profile happens within 60–100 days before a steady-state is reached. In the current experiment, a reduction of EPA + DHA had taken place from the beginning of the experiment to the end after 56 days. During this time, fish held at 23 °C tripled their weight, whereas fish held at 19 °C only doubled their weight. Simultaneously, the final whole body and fillet tissue EPA + DHA contents were lower in fish held at 23 °C. Within the dietary treatment groups held at 23 °C, the fish fed the fish oil-reduced diets had significantly lower final EPA + DHA levels compared to those that were fed the control diet. Thus, the dilution of the previous tissue EPA + DHA content was more severe in fish with a faster growth that were fed extremely low amounts of EPA + DHA. Other environmentally triggered mechanisms affecting the FA composition of tissues in order to maintain functionality of physiological processes [48] might have occurred simultaneously but cannot be clearly determined. 

The supplementation of RV to the diet with 6% DM fish oil led to a significant increase in the liver EPA + DHA content. These LC-PUFA increasing effects of RV had been previously reported for rat hepatocytes [31], zebrafish embryos [17], and rainbow trout [15]. The possible underlying mechanisms reported by the literature include an increase in the abundance or activity of the desaturases [15,31] or the protection of FAs from oxidation by RV [32]. On the contrary, RV supplementation to the diet with 2% DM fish oil led to a decrease in the EPA + DHA content in the sea bream livers. It is not fully clear why RV acted differently in the various dietary treatments. In our previous study with rainbow trout, we already observed that RV affects the tissue PUFA levels dependent on the dietary fish oil level [15]. In addition, Ran et al. [49] described a bi-directional interaction of RV with the lipid metabolism of zebrafish. It should be taken into account that RV may interact with different pathways affecting the FA composition. First of all, RV can putatively influence the uptake of FAs into liver tissue [50]. Further, RV is a potent influencer of the lipid metabolism, for example, via the hepatic sirtuin pathway [26]. If such effects combined with the differing dietary FA composition and different substrates for these pathways play a role in the RV-mediated responses remains to be elucidated. Whether the differences in the response of the fish to dietary RV can be associated with the difference in species, the difference in lifestyle (freshwater vs. marine and the concomitant differences in the ability to perform the FA synthesis [13,24]), or other factors cannot be concluded from such few studies yet.

### 3.4. mRNA Steady-State Levels of Genes Related to Fatty Acid Synthesis and Metabolism

The mRNA steady-state levels of the ∆6-D gene and genes involved in the FA metabolism and β-oxidation were significantly affected by the dietary fish oil level and also partly by the dietary RV-supplementation. A diet-induced up-regulation of the mRNA expression and enzyme activity of the ∆6-D, a key enzyme in FA synthesis had been observed in marine fish fed PUFA-deficient diets, for example, sea bream [20,51] and sea bass [21]. The results of the present study support these previous findings of the upregulation of the mRNA expression of ∆6-D when sea bream are fed diets low in fish oil. Furthermore, the increase in the PPARα mRNA steady-state levels caused by the change in the dietary fatty acid composition could have been mediated by the dietary PUFAs and MUFAs, which are effective substrates for PPARs [52]. It has been shown that the nutritional status of fish influences the mRNA expression of PPARα [53], a transcription factor involved in many aspects of the hepatic lipid metabolism including the FA uptake, FA activation, and FA oxidation [54]. In this study, the hepatic mRNA steady-state levels of ECH and CEL were increased by low dietary fish oil levels. A dietary induced regulation of the hepatic mRNA expression of ECH and CEL had been suggested previously for sea bream [55,56] and sea bass [57]. An interaction between the dietary fish oil and PUFA content and expression of ECH and CEL seems likely since ECH is involved in the mitochondrial β-oxidation of FA [56] and CEL is involved in the lipid and FA uptake, transport, and metabolism [58].

In this study, the RV-mediated modification of the EPA + DHA levels in livers of sea bream can be (partly) explained by the differences in the mRNA steady-state levels of ∆6-D and PPARα. The increased hepatic ∆6-D mRNA steady-state level of fish fed the control diet supplemented with RV might explain the simultaneously increased EPA + DHA content in the fish livers. This is supported by previous findings in rat hepatocytes, where the RV interacted with the ∆6- and ∆5-desaturases, thus, increasing the LC-PUFA content [31]. The decreased hepatic PPARα mRNA steady-state levels in fish fed the fish oil-reduced diet supplemented with RV might be a possible explanation for the concomitant decrease in the tissue EPA + DHA content. The RV-mediated reduced steady-state level of the PPARα-gene is controversial as compared to previous studies in which RV was described as a PPAR activator (reviewed by Nakata et al. [59]), or where RV had no effect on the PPARα expression [15,17]. We assume that other mechanisms and molecular pathways might have been modified by RV and influenced the FA composition in sea bream liver tissue [26,32,49,60]. Nevertheless, with the results obtained in this study, we can expand the knowledge concerning the genetic capacity of sea bream to perform an endogenous FA synthesis. The dietary oil source not only influences the tissue LC-PUFA levels, but also affects genes involved in the FA synthesis and metabolism. The role of RV in the nutritional regulation of the mRNA steady-state levels of ∆6-D and PPARα still needs to be clarified. From this study it cannot be fully concluded whether or not RV stimulated the FA synthesis. No enzyme activities were measured and no positive FA retention could be observed throughout the experimental trial.

## 4. Materials and Methods

### 4.1. Experimental Diets

Four different experimental diets (isonitrogenous and isoenergetic) were formulated as shown in Table 7. All diets consisted of mainly alternative plant protein sources (Soybean concentrate, corn gluten, wheat gluten, and rapeseed expeller) and had a fish meal content of 5% DM. Diet F6, the overall control diet, contained 6% DM fish oil and 3% DM of a mixture of vegetable oils (linseed, rapeseed, and palm oil). Diet F2 had a fish oil content of 2% DM and contained 7% DM of a mixture of linseed, rapeseed, and palm oil. The diets contained considerably different amounts of the fatty acids EPA and DHA as shown in Table 3. Control diet F6 was formulated to meet the recommended amount of EPA and DHA for sea bream (EPA + DHA: 0.9% of dry diet when DHA/EPA = 1 [40,41]). Diet F2 contained reduced amounts of EPA and DHA (0.37% of dry diet, Table 3) below the recommendation for sea bream. Experimental diets F6 + RV and F2 + RV were equal to diets F6 and F2, respectively, and supplemented with resveratrol (+ RV; *trans*-3,4',5-trihydroxy stilbene, purity ≥ 98%, Chemos GmbH & Co. KG, Regenstauf, Germany) at a concentration of 0.15% DM of the diet. The dietary essential amino acid content of each diet was calculated from the amino acid content of the single ingredients. All diets were formulated based on the macronutrient and the essential amino acid requirements of gilthead sea bream according to Peres and Olivia-Teles [61] and Wilson [62]. All diets were pressed into 3- and 4-mm pellets using a feed press L14-175 (Amandus Kahl, Reinbek, Germany).

### 4.2. Experimental Setup

The study was conducted at the facilities of the Grupo de Investigación en Acuicultura, Universidad de Las Palmas de Gran Canaria (GIA-ULPGC), Telde, Las Palmas, Canary Islands, Spain. All experiments were carried out according to the EU Directive 2010/63/EU for animal experiments and approved by the Ministry of Energy, Agriculture, the Environment, Nature and Digitalization (MELUND), Kiel, Germany (approved on 15th October 2014; project number: V244-7224.121.9-34). Two experimental setups were prepared for the feeding trial to realize a simultaneous diet- and temperature-challenge. (1) A flow-through system with 12 tanks was used for the high-temperature challenge (approx. 23 °C). (2) Identical temperature-controlled small recirculating aquaculture systems equipped with a total of 12 tanks were used for the low-temperature challenge (approx. 19 °C).

(1) The flow-through system had a natural photoperiod (12 h light) during the whole adaptation and experimental period. The cylindrical fiberglass tanks (500 L) were supplied with filtered seawater (37‰ salinity) at a rate of 600 L h^−1^ and were continuously aerated. Temperature (22.6 ± 0.6 °C), oxygen (6.2 ± 0.3 mg L^−1^), and pH (8.22; pH-Meter Basic 20+, CRISON, Hach Lange Spain, Barcelona, Spain) were monitored daily. Ammonia (NH_4_: < 0.15) and nitrite (NO_2_: < 0.1, Royal Ammonia Professional Test and Royal Nitrite Professional Test, Royal Nature, Nesher, Israel) concentrations were determined every second day. 

(2) The recirculating systems (kept at a photoperiod of 12 h light) had 3000 L of water volume each and were equipped with a cooling unit and a water treatment unit consisting of a sand filter (filtration area: 0.33 m^3^; granular size: 0.4–1.2 mm, AstralPool, Fluidra, Barcelona, Spain), a multibed bio filter (filtration area: 0.32 m^2^; Kripsol, Toledo, Spain), and a protein skimmer. Each recirculating system had three cylindrical fiberglass tanks (500 L) that were supplied with temperature controlled sea water, the same as in the flow-through system. The water quality parameters did not differ between the systems and were measured as described above (temperature: 19.0 ± 1.5 °C; oxygen: 7.1 ± 0.3 mg L^−1^ O_2_; pH: 8.06; NH_4_: <1; NO_2_: <0.5).

A total of 600 juvenile gilthead sea bream (offspring from brood stock of GIA-ULPGC, initial body weight: 12.5 ± 2.2 g) were acclimated in the flow-through system and randomly and equally distributed among all 24 tanks of both setups, 25 individuals per tank. Over the first seven days of the experimental period, the recirculating systems were continuously cooled down from 23 °C to 19 °C. The four dietary treatments (F6, F6 + RV, F2, and F2 + RV) were randomly distributed among the twelve tanks of each setup, ensuring that each dietary treatment was tested at each temperature in a triplicate approach. During the whole experimental period of 55 days, the fish were fed manually three times per day until apparent satiation in both setups. The administered feed ratios were determined daily for the calculation of the DFI.

### 4.3. Sampling

The tissue samples were collected before the onset of the feeding trial (day 0) and at the end of the trial (day 56). For the initial sampling at day 0, seven fish were sacrificed (pooled sample) and stored at −80 °C for the determination of the whole body nutrient composition and fatty acid composition. Additionally, six fish were sacrificed for the collection of liver and fillet tissue samples. The whole liver was weighed (±0.01 g) for the determination of the HSI (see Equation (1)). For mRNA quantification via quantitative real-time RT-PCR, one part of the liver tissue was preserved in RNALater (Sigma-Aldrich, Taufkirchen, Germany). For the determination of the liver fatty acid composition, the remaining liver parts of all six individuals were pooled in one sample and immediately frozen at −80 °C. The fillets (left side) from all six fish were pooled and frozen at −80 °C for the determination of the fatty acid composition. For the final sampling at day 56, five fish per tank were sacrificed (pooled sample) and stored at −80 °C for the determination of the whole body nutrient composition and the fatty acid composition. Additionally, five fish per tank were sacrificed for the collection of the liver and fillet tissue samples. The procedure was the same as described for the initial sampling. 

For the determination of the growth, performance, and nutrient utilization parameters, all fish were individually weighed (±0.1 g) and measured (±0.1 cm) at day 0 and day 56 for the calculation of the initial and final body weights (IBW and FBW, respectively). The SGR, FCR, PER, PPV, and FCF were calculated according to the Equations (2)–(6).

(1) $$\mathrm{HSI}\;\left[\%\right]=\frac{\mathrm{liver}\;\mathrm{weight}\;\left[g\right]}{\mathrm{FBW}\;\left[g\right]}\times 100$$

(2) $$\mathrm{SGR}\;\left[{\%\;d}^{-1}\right]=\frac{\left[\ln\;\left(\mathrm{FBW}\right)-\ln\;\left(\mathrm{IBW}\right)\right]}{\mathrm{feeding}\;\mathrm{day}}\times 100$$

(3) $$\mathrm{FCR}=\frac{\mathrm{feed}\;\mathrm{intake}\;\left[g\right]}{\mathrm{weight}\;\mathrm{gain}\;\left[g\right]}$$

(4) $$\mathrm{PER}=\frac{\mathrm{weight}\;\mathrm{gain}\;\left[g\right]}{\mathrm{protein}\;\mathrm{intake}\;\left[g\right]}$$

(5) $$\mathrm{PPV}\;\left[\%\right]=\frac{\left(\mathrm{final}\;\mathrm{body}\;\mathrm{protein}\;\left[g\right]\times \mathrm{FBW}\;\left[g\right]\right)-\left(\mathrm{initial}\;\mathrm{body}\;\mathrm{protein}\;\left[g\right]\times \mathrm{IBW}\;\left[g\right]\right)}{\mathrm{protein}\;\mathrm{intake}\;\left[g\right]}\times 100$$

(6) $$\mathrm{FCF}=100\times \left(\mathrm{FBW}\;\left[g\right]\times \mathrm{final}\;\mathrm{body}\;\mathrm{length}{\left[\mathrm{cm}\right]}^{-3}\right)$$

### 4.4. Nutrient Composition Analysis

The nutrient composition was analyzed in all experimental diets and the whole body homogenates of the gilthead sea bream. Frozen whole body samples were freeze-dried (Alpha 1,2 LDplus and Alpha 1–4 LSC, Martin Christ Gefriertrocknungsanlagen GmbH, Osterode am Harz, Germany) until the weight was stable and homogenized using a cutting mill (GM 200, Retsch, Haan, Germany). Diets were homogenized using a mortar and pestle. Analysis of nutrients and gross energy was done according to the EU guideline (EC) 152/2009 [63] as described in Torno et al. [46]. The total carbohydrates were calculated according to formula (7):

(7) Total carbohydrates=1000−(crude protein+crude lipid+crude ash)

### 4.5. Lipid Extraction and Fatty Acid Composition Analysis

The total lipids were extracted from the whole body homogenates, liver samples, fillet samples, and diet samples according to Folch et al. [64]. The measurement of fatty acid methyl esters (FAMEs) was performed using a gas chromatograph with a flame ionization detector (Agilent Technologies, Santa Clara, CA, USA). A detailed description is given in Torno et al. [15]. A 13-FAME standard was used to identify the retention times of the individual FAMEs (Table 4, Table 5 and Table 6). The fatty acid composition was calculated as a percentage of a single FAME relative to the total FAMEs. The internal standard 13:0 methyl ester was used to calculate FAs as a % DM of the diet.

### 4.6. mRNA Extraction and Quantitative Real-Time RT-PCR

Based on the results of growth, performance, and FA composition measurement of the mRNA steady-state levels was performed only in the liver of fish held at 19 °C. The total mRNA was extracted from the liver samples using the Innuprep RNA Mini Kit (Analytik Jena, Jena, Germany) according to the manufacturer’s protocol as described in Torno et al. [15]. The primers used and the appropriate annealing temperatures are listed in Table 8. The transcript expression was quantified by calculating the input copy number using a standard curve. Subsequently, the respective target mRNA steady-state levels of ∆6-D, PPARα, ECH and CEL were normalized to the mRNA levels of the housekeeping gene beta-actin (β-actin). Data are shown as relative mRNA steady-state levels of respective target genes normalized to their internal control (β-actin) following absolute quantification (Figure 1).

### 4.7. Statistical Analysis

All statistical analyses were performed using R (version 3.1.3) with an RStudio interface. The packages *gdata*, *multcomp*, *gplots*, *nparcomp*, *nlme*, *piecewiseSEM*, *SimComp*, and *car* were used for the graphical and the statistical analysis.

The data evaluation started with the definition of an appropriate statistical model based on a graphical residual analysis of the data and Levene’s test to test for the homoscedasticity of variances: (1) statistical model based on generalized least squares (*gls*) for normally distributed and heteroscedastic data (IBW, FCR, crude protein) and (2) linear model (*lm*) for normally distributed and homoscedastic data (DFI, FBW, FCF, HIS, SGR, PER, PPV, crude ash, crude fat, gross energy, dry matter, and fatty acid composition). Both models included the level of dietary fish oil content (6% and 2% DM), supplement (None, RV), and temperature (19 °C, 23 °C), as well as their interaction term as fixed factors. An analysis of variances (ANOVA) was conducted followed by an appropriate post-hoc test: (1) ANOVA based on *gls* was followed by multiple contrast tests for heteroscedastic data according to Hasler and Hothorn [65]; (2) ANOVA based on *lm* was followed by multiple contrast tests according to Schaarschmidt and Vaas [66]. All post-hoc tests compared the feeding groups based on the fish oil level and supplement within one holding temperature and between the two temperatures.

For mRNA steady-state levels (∆6-D, PPARα, ECH, CEL) the data evaluation was initiated with the definition of an appropriate mixed model (*lme*) with a fish oil content (6% and 2% DM), supplement (None, RV) and their interaction term as fixed factors, and fish tank as random factor. A residual analysis revealed the data to be non-normally distributed. Multiple contrast tests for relative effects were conducted in order to compare the influence of varying factors [66].

## 5. Conclusions

The present study has demonstrated that it is possible to feed juvenile sea bream with diets that are very low in fish meal (5% DM) and fish oil (2% DM) without negatively affecting their growth performance or nutrient utilization. However, the fish oil reduction in the diets from 6% to 2% DM lead to a decrease in the whole body crude lipid, gross energy, relative PUFA content, and relative EPA and DHA content, despite the promoted up-regulation of ∆6-D, PPARα, ECH, and CEL. An increase in water temperature from 19 °C to 23 °C markedly increased the feed intake and the growth performance in sea bream, but decreased the tissue EPA + DHA contents. On the contrary, the supplementation of RV increased the EPA + DHA content in fish liver tissue most likely via an up-regulation of ∆6-D, particularly in seabream fed 6% DM fish oil. Further studies need to be conducted to clarify the potential value of RV in fish diets to enhance the fillet EPA + DHA contents.

## Figures and Tables

**Table 1:** The growth, nutrient utilization, performance, and final whole body nutrient composition (percentage of original matter (% OM) and MJ kg^−1^ OM) of gilthead sea bream fed with the experimental diets for 8 weeks and held at two different temperatures, 19 °C and 23 °C. F6 and F2 indicate the feeding with the basal diets containing 6% and 2% dry matter (DM) fish oil, respectively. +RV indicates feeding the diets supplemented with 0.15% DM resveratrol.

Performance Parameter	19 °C				23 °C				Comparison between 19 and 23 °C			
	F6	F2	F6 + RV	F2 + RV	F6	F2	F6 + RV	F2 + RV	F6	F2	F6 + RV	F2 + RV
IBW ^1^	12.4 ± 0.3	12.5 ± 0.3	12.5 ± 0.3	12.8 ± 0.4	12.6 ± 0.2	12.2 ± 0.2	12.7 ± 0.2	12.5 ± 0.1				
FBW ^2^	29.7 ± 0.9	27.0 ± 1.7	28.4 ± 1.1	26.0 ± 0.6	42.3 ± 1.3	40.4 ± 0.9	44.1 ± 1.5	40.0 ± 5.0	***	***	***	***
SGR ^3^	1.6 ± 0.1	1.4 ± 0.1	1.5 ± 0.0	1.3 ± 0.0	2.3 ± 0.1	2.3 ± 0.0	2.4 ± 0.1	2.2 ± 0.2	***	***	***	***
DFI ^4^	3.2 ± 0.2	3.0 ± 0.2	3.3 ± 0.2	3.2 ± 0.3	4.1 ± 0.2	4.3 ± 0.1	4.4 ± 0.0	4.3 ± 0.2	***	***	***	***
FCR ^5^	2.1 ± 0.2	2.2 ± 0.1	2.2 ± 0.2	2.5 ± 0.2	1.8 ± 0.0	1.9 ± 0.0	1.9 ± 0.1	1.9 ± 0.1				
PER ^6^	1.1 ± 0.1	0.9 ± 0.2	1.0 ± 0.1	0.9 ± 0.1	1.2 ± 0.1	1.1 ± 0.1	1.2 ± 0.1	1.1 ± 0.1				(*)
PPV ^7^	16.7 ± 1.8	14.3 ± 3.1	14.9 ± 1.4	13.6 ± 1.5	18.5 ± 1.4	17.7 ± 0.6	17.9 ± 0.7	17.1 ± 1.0		(*)		(*)
HSI ^8^	2.4 ± 0.3	2.7 ± 0.1	2.2 ± 0.1 ^n^	2.7 ± 0.2 ^m^	2.0 ± 0.1	2.1 ± 0.2	1.7 ± 0.1	2.0 ± 0.3	*	*	*	*
FCF ^9^	1.6 ± 0.0	1.6 ± 0.0	1.5 ± 0.0	1.5 ± 0.0	1.7 ± 0.0	1.7 ± 0.0	1.7 ± 0.0	1.7 ± 0.1	*	*	*	*
Nutrient Composition (% OM)												
Dry matter	30.9 ± 0.8	29.6 ± 0.6	29.3 ± 0.2	30.2 ± 0.3	33.5 ± 1.8	32.4 ± 0.6	32.8 ± 1.3	32.1 ± 0.7	*	**	**	(*)
Crude ash	3.8 ± 0.0	3.9 ± 0.1	3.9 ± 0.1	3.9 ± 0.1	3.4 ± 0.1 ^(b),(B)^	3.6 ± 0.1 ^(a)^	3.6 ± 0.1 ^(A)^	3.8 ± 0.1	***	**	**	
Crude protein	16.0 ± 0.2	16.2 ± 0.1	15.9 ± 0.2	16.2 ± 0.3	16.0 ± 0.8	16.3 ± 0.1	15.9 ± 0.4	16.2 ± 0.4				
Crude lipid	10.7 ± 0.8 ^(a),(A)^	9.2 ± 0.7 ^(b)^	9.1 ± 0.2 ^(B)^	9.7 ± 0.4	14.1 ± 0.9 ^a^	12.4 ± 0.9 ^b^	13.2 ± 0.7	12.1 ± 0.9	***	***	***	**
Gross energy ^10^	8.07 ± 0.32 ^(A)^	7.44 ± 0.28	7.32 ± 0.15 ^(B)^	7.64 ± 0.12	9.31 ± 0.54	8.73 ± 0.31	9.03 ± 0.49	8.60 ± 0.34	**	**	***	*

^1^ IBW = Initial body weight (g); ^2^ FBW = Final body weight (g); ^3^ SGR = Specific growth rate (% d^−1^); ^4^ DFI = Daily feed intake (% d^−1^); ^5^ FCR = Feed conversion ratio; ^6^ PER = Protein efficiency ratio; ^7^ PPV = Protein productive value (%); ^8^ HSI = Hepatosomatic index (%); ^9^ FCF = Fulton condition factor; ^10^ Gross energy is given in MJ kg^−1^ OM. Initial nutrient composition: dry matter: 29.7%; crude ash: 4.2% OM; crude protein: 17.0% OM; crude lipid: 8.4% OM; gross energy: 7.34 MJ kg^−1^ OM. Values (mean ± SD, n = 3; HSI, SSI, and FCF: n = 15) with different superscript letters and different types of letters within one temperature treatment differ with *p*-values < 0.05 based on ANOVA, as described in the Materials and Methods section. The superscript letters indicate the output of the tests based on comparisons of the fish oil level within one supplement group (a, b: F6 vs. F2; m, n: F6 + RV vs. F2 + RV) and the effect of supplementation within one fish oil level group (A, B: F6 vs. F6 + RV) separated by temperature. The statistical outputs of the test based on the effect of the different holding temperatures analyzed within one feeding group are indicated in separate columns using * for *p* < 0.05, ** for *p* < 0.01, and *** for *p* < 0.001. All designations in brackets indicate a tendency towards a difference based on *p* < 0.1.

**Table 2:** The statistic significance of effects (*p*-values) caused by the dietary fish oil level (Oil), resveratrol supplementation (RV), holding temperature (Temp.), and the interaction of either two factors or all three factors. The *p*-values are given for the effects on the selected growth and performance parameters, body nutrients, and fatty acids (FAs) of different tissues of gilthead sea bream at the end of the eight weeks trial.

	Responding Parameter	Single Effects						Interactions							
		Oil		RV		Temp.		Oil:RV		Oil:Temp.		RV:Temp.		Oil:RV:Temp.	
Growth and Performance	FBW ^1^	0.005	**	0.811		<0.001	***	0.573		0.786		0.298		0.443	
	SGR ^2^	0.003	**	0.280		<0.001	***	0.427		0.254		0.236		0.477	
	DFI ^3^	0.461		0.182		<0.001	***	0.470		0.220		0.939		0.082	(*)
	HSI ^4^	<0.001	***	0.047	*	<0.001	***	0.141		0.280		0.612		0.630	
	FCF ^5^	0.371		0.078	(*)	<0.001	***	0.981		0.514		0.398		0.468	
	Dry matter	0.161		0.200		<0.001	***	0.094	(*)	0.378		0.961		0.245	
	Crude ash	0.014	*	0.006	**	<0.001	***	0.201		0.127		0.233		0.522	
	Crude lipid	0.007	**	0.076	(*)	<0.001	***	0.045	*	0.123		0.916		0.195	
	Gross energy	0.031	*	0.111		<0.001	***	0.069	(*)	0.233		0.817		0.174	
Whole Body FAs	Σ SFA ^6^	<0.001	***	0.036	*	<0.001	***	0.844		0.489		0.337		0.479	
	Σ MUFA ^7^	<0.001	***	0.054	(*)	0.001	***	0.018	*	0.002	**	0.961		0.147	
	Σ PUFA ^8^	0.003	**	0.027	*	<0.001	***	0.130		0.058	(*)	0.666		0.536	
	EPA + DHA ^9^	<0.001	***	0.136		<0.001	***	0.742		<0.001	***	0.368		0.756	
Liver Tissue FAs	Σ SFA	0.029	*	0.583		<0.001	***	0.668		0.890		0.201		0.650	
	Σ MUFA	0.001	**	0.377		0.080	(*)	0.008	**	0.093	(*)	0.762		0.451	
	Σ PUFA	0.105		0.317		<0.001	***	0.026	*	0.193		0.266		0.398	
	EPA + DHA	<0.001	***	0.534		<0.001	***	0.003	**	0.583		0.811		0.018	*
Fillet tissue FAs	Σ SFA	<0.001	***	0.179		<0.001	***	0.364		0.053	(*)	0.587		0.428	
	Σ MUFA	<0.001	***	0.130		0.053	(*)	0.988		0.267		0.237		0.639	
	Σ PUFA	0.007	**	0.061	(*)	<0.001	***	0.679		0.073	(*)	0.205		0.450	
	EPA + DHA	<0.001	***	0.221		<0.001	***	0.720		0.019	*	0.219		0.270	

^1^ FBW = Final body weight, ^2^ SGR = Specific growth rate, ^3^ DFI = Daily feed intake, ^4^ HSI = Hepatosomatic index, ^5^ FCF = Fulton condition factor, ^6^ SFA = Saturated fatty acids, ^7^ MUFA = Monounsaturated fatty acids, ^8^ PUFA = Polyunsaturated fatty acids, ^9^ EPA+DHA = Sum of eicosapentaenoic acid (EPA) and docosahexaenoic acid (DHA). Significant effects are indicated with * (*p* < 0.5), ** (*p* < 0.01), and *** (*p* < 0.001). A statistical tendency (*p* < 0.1) is indicated by (*).

**Table 3:** The fatty acid composition (in the % of total fatty acid methyl esters (FAMEs) and the % of dry matter of diet (% DM)) of the experimental diets. F6 and F2 are the basal diets containing 6% and 2% dry matter (DM) fish oil, respectively. +RV indicates the supplementation of the basal diets with 0.15% DM resveratrol. The standard used for the identification of individual FAMEs consisted of all 12 FAMEs shown here.

% of FAMEs	F6	F2	F6 + RV	F2 + RV
14:0	2.66	1.31	2.58	1.30
16:0	19.29	27.82	19.41	27.98
18:0	3.60	3.74	3.56	3.74
**Σ SFA ^1^**	**25.55**	**32.87**	**25.54**	**33.02**
16:1n-7	2.83	1.16	2.67	1.13
18:1n-7c	2.51	1.90	2.44	1.90
18:1n-9c	24.54	24.72	24.11	24.73
**Σ MUFA ^2^**	**29.88**	**27.78**	**29.22**	**27.76**
18:2n-6c (LA) ^3^	24.80	24.19	25.24	24.20
18:3n-3 (ALA) ^4^	11.77	12.11	12.48	12.03
18:3n-6	0.15	0.05	0.13	0.05
20:5n-3 (EPA) ^5^	3.13	1.17	3.00	1.18
22:5n-3	0.77	0.26	0.70	0.27
22:6n-3 (DHA) ^6^	3.89	1.58	3.63	1.50
**Σ PUFA ^7^**	**44.52**	**39.35**	**45.20**	**39.22**
**Σ EPA + DHA**	**7.02**	**2.75**	**6.63**	**2.68**
DHA/EPA	1.24	1.35	1.21	1.27
ALA/LA	0.47	0.50	0.49	0.50
**Σ EPA + DHA % DM ^8^**	0.95	0.37	0.88	0.37

^1^ Σ SFA is the sum of saturated fatty acids; ^2^ Σ MUFA is the sum of the monounsaturated fatty acids; ^3^ LA: Linoleic acid; ^4^ ALA: α-Linolenic acid; ^5^ EPA: Eicosapentaenoic acid; ^6^ DHA: Docosahexaenoic acid; ^7^ Σ PUFA is the sum of the n-3 and n-6 polyunsaturated fatty acids; ^8^ The determination of EPA + DHA % DM of diet was done using the internal standard 13:0 methyl ester and amount of lipid measured in the diet (Table 7).

**Table 4:** The fatty acid composition (percentage of total fatty acid methyl esters (% FAMEs)) of whole body homogenates of gilthead sea bream at the end of the 8-week feeding trial held at two different temperatures (19 °C and 23 °C). F6 and F2 indicate the feeding with the basal diets containing 6% and 2% dry matter (DM) fish oil, respectively. +RV indicates the feeding diets supplemented with 0.15% DM resveratrol.

% FAMEs	19 °C				23 °C				Comparison between 19 °C and 23 °C			
	F6	F2	F6 + RV	F2 + RV	F6	F2	F6 + RV	F2 + RV	F6	F2	F6 + RV	F2 + RV
14:0	2.8 ± 0.1 ^a^	2.5 ± 0.1 ^b^	2.7 ± 0.1 ^(m)^	2.5 ± 0.2 ^(n)^	2.8 ± 0.1 ^a^	2.3 ± 0.1 ^b^	2.8 ± 0.1 ^m^	2.2 ± 0.2 ^n^				*
16:0	17.2 ± 0.2	16.8 ± 0.2	17.0 ± 0.5	16.5 ± 0.1	18.9 ± 0.2	18.6 ± 0.5	18.7 ± 0.2	18.2 ± 0.4	***	***	***	***
18:0	4.4 ± 0.2	4.6 ± 0.1	4.6 ± 0.2	4.5 ± 0.0	5.1 ± 0.2 ^A^	4.9 ± 0.1	4.7 ± 0.1 ^B^	4.9 ± 0.1	***	*		**
**Σ SFA ^1^**	**24.4 ± 0.4**	**23.9 ± 0.2**	**24.3 ± 0.6**	**23.5 ± 0.1**	**26.9 ± 0.4 ^a^**	**25.9 ± 0.5 ^b^**	**26.2 ± 0.3**	**25.4 ± 0.5**	***	***	***	***
14:1n-5	0.1 ± 0.0	0.1 ± 0.0	0.1 ± 0.0	0.1 ± 0.0	0.1 ± 0.0	0.1 ± 0.0	0.1 ± 0.0	0.1 ± 0.0				
16:1n-7	4.4 ± 0.2 ^a^	3.9 ± 0.1 ^b^	4.2 ± 0.1	4.0 ± 0.3	4.5 ± 0.1 ^a^	3.7 ± 0.2 ^b^	4.5 ± 0.2 ^m^	3.6 ± 0.2 ^n^				
18:1n-7c	3.1 ± 0.1	3.0 ± 0.0	3.1 ± 0.1	3.0 ± 0.1	2.9 ± 0.1 ^a^	2.6 ± 0.0 ^b^	2.9 ± 0.0 ^m^	2.7 ± 0.1 ^n^	**	***	***	***
18:1n-;9c	32.0 ± 0.7 ^A^	32.8 ± 0.3	30.6 ± 1.0 ^B,n^	33.1 ± 0.6 ^m^	31.6 ± 0.4 ^b^	35.5 ± 0.4 ^a^	31.1 ± 0.3 ^n^	35.3 ± 0.4 ^m^		***		***
**Σ MUFA ^2^**	**39.5 ± 0.9 ^A^**	**39.7 ± 0.2**	**38.1 ± 0.9 ^B,n^**	**40.2 ± 0.5 ^m^**	**39.2 ± 0.1 ^b^**	**41.8 ± 0.6 ^a^**	**38.5 ± 0.2 ^n^**	**41.6 ± 0.7 ^m^**		**		*
18:2n-6c	18.8 ± 0.6	19.5 ± 0.2	19.3 ± 0.5	19.3 ± 0.6	17.7 ± 0.3	18.6 ± 0.7	18.5 ± 0.2	18.9 ± 0.8	(*)			
18:3n-3	7.5 ± 0.3	7.6 ± 0.2	7.7 ± 0.2	7.4 ± 0.5	7.4 ± 0.0	8.0 ± 0.4	8.0 ± 0.2	8.2 ± 0.5				*
18:3n-6	0.9 ± 0.1 ^B,b^	1.2 ± 0.0 ^a^	1.1 ± 0.1 ^A^	1.2 ± 0.1	0.8 ± 0.0	0.9 ± 0.1	0.8 ± 0.1 ^n^	1.0 ± 0.1 ^m^		***	***	**
20:5n-3 (EPA) ^3^	3.0 ± 0.1	2.7 ± 0.2	3.2 ± 0.3	2.9 ± 0.4	2.9 ± 0.1 ^a^	1.7 ± 0.1 ^b^	2.9 ± 0.1 ^m^	1.7 ± 0.2 ^n^		***		***
22:5n-3	1.3 ± 0.1	1.2 ± 0.1	1.3 ± 0.1	1.2 ± 0.1	1.1 ± 0.0 ^a^	0.7 ± 0.0 ^b^	1.1 ± 0.1 ^m^	0.7 ± 0.1 ^n^	*	***	**	***
22:6n-3 (DHA) ^4^	4.6 ± 0.2	4.2 ± 0.3	5.0 ± 0.4 ^m^	4.4 ± 0.5 ^n^	4.0 ± 0.0 ^a^	2.5 ± 0.1 ^b^	4.2 ± 0.2 ^m^	2.5 ± 0.2 ^n^	(*)	***	**	***
**Σ PUFA ^5^**	**36.1 ± 1.1**	**36.4 ± 0.4**	**37.6 ± 1.5**	**36.3 ± 0.5**	**34.0 ± 0.3**	**32.3 ± 1.1**	**35.3 ± 0.2 ^m^**	**33.0 ± 1.0 ^n^**	*	***	*	**
EPA + DHA	7.7 ± 0.3	6.9 ± 0.5	8.2 ± 0.7 ^(m)^	7.3 ± 0.8 ^(n)^	6.9 ± 0.1 ^a^	4.1 ± 0.1 ^b^	7.0 ± 0.3 ^m^	4.3 ± 0.3 ^n^		***	*	***
EPA/DHA	0.7 ± 0.0	0.6 ± 0.0	0.6 ± 0.0	0.7 ± 0.0	0.7 ± 0.0	0.7 ± 0.0	0.7 ± 0.0	0.7 ± 0.0				
n-3/n-6	0.8 ± 0.0	0.8 ± 0.0	0.9 ± 0.0	0.8 ± 0.0	0.8 ± 0.0	0.7 ± 0.0	0.8 ± 0.0	0.7 ± 0.0				

^1^ Σ SFA is the sum of the saturated fatty acids; ^2^ Σ MUFA is the sum of the monounsaturated fatty acids; ^3^ EPA: Eicosapentaenoic acid; ^4^ DHA: Docosahexaenoic acid; ^5^ Σ PUFA is the sum of n-3 and n-6 polyunsaturated fatty acids. The values (mean ± SD, *n* = 3) with different superscript letters and different types of letters within one temperature treatment differ with *p*-values < 0.05 based on ANOVA, as described in the Materials and Methods section. The superscript letters indicate the output of tests based on comparisons of fish oil level within one supplement group (a, b: F6 vs. F2; m, n: F6 + RV vs. F2 + RV) and the effect of supplementation within one fish oil level (A, B: F6 vs. F6 + RV), separated by temperature. The statistical outputs of the test based on the effect of the different holding temperatures analyzed within one feeding group are indicated in separate columns using * for *p* < 0.05, ** for *p* < 0.01 and *** for *p* < 0.001. All designations in brackets indicate a tendency towards a difference based on *p* < 0.1.

**Table 5:** The fatty acid composition (percentage of total fatty acid methyl esters (% FAMEs)) of liver tissue of gilthead sea bream at the end of the 8-week feeding trial held at two different temperatures (19 °C and 23 °C). F6 and F2 indicate the feeding with the basal diets containing 6% and 2% dry matter (DM) fish oil, respectively. +RV indicates the feeding diets supplemented with 0.15% DM resveratrol.

% FAMEs	19 °C				23 °C				Comparison between 19 °C and 23 °C			
	F6	F2	F6 + RV	F2 + RV	F6	F2	F6 + RV	F2 + RV	F6	F2	F6 + RV	F2 + RV
14:0	1.7 ± 0.1	1.7 ± 0.1	1.6 ± 0.1	1.6 ± 0.1	2.2 ± 0.3	2.2 ± 0.2	2.1 ± 0.2	2.2 ± 0.1	**	**	**	***
16:0	15.5 ± 0.6	14.1 ± 0.8	14.4 ± 1.2	14.4 ± 0.9	19.4 ± 0.8	18.4 ± 0.4	20.1 ± 0.6	19.4 ± 0.2	***	***	***	***
18:0	6.4 ± 0.5	6.4 ± 0.4	6.9 ± 0.8	6.3 ± 0.6	7.8 ± 0.0	7.6 ± 0.2	8.0 ± 0.0	7.5 ± 0.6	*	*	*	*
**Σ SFA ^1^**	**23.6 ± 1.0**	**22.2 ± 1.2**	**22.9 ± 2.0**	**22.2 ± 1.4**	**29.4 ± 0.9**	**28.2 ± 0.7**	**30.3 ± 0.8**	**29.1 ± 0.6**	***	***	***	***
14:1n-5	0.0±0.0	0.0±0.0	0.0±0.0	0.0±0.0	0.0±0.0	0.0±0.0	0.0±0.0	0.0±0.0				
16:1n-7	3.8 ± 0.1 ^a^	3.1± 0.1 ^b^	3.4 ± 0.0 ^m^	2.9 ± 0.3 ^n^	3.8 ± 0.1 ^a^	3.2 ± 0.2 ^b^	4.0 ± 0.1 ^m^	3.5 ± 0.3 ^n^			**	**
18:1n-7c	3.0 ± 0.1	2.9 ± 0.0	3.0 ± 0.1	2.9 ± 0.0	2.8 ± 0.1	2.6 ± 0.1	2.9 ± 0.1 ^m^	2.6 ± 0.2 ^n^	(*)	*		**
18:1n-9c	39.0 ± 0.7	41.1 ± 0.5	37.8 ± 1.8 ^n^	43.4 ± 1.5 ^m^	40.3 ± 1.9	41.5 ± 0.3	39.6 ± 0.7 ^n^	42.9 ± 0.8 ^m^				
**Σ MUFA ^2^**	**45.8 ± 0.6**	**47.1 ± 0.4**	**44.3 ± 1.7 ^n^**	**49.2 ± 1.7 ^m^**	**47.0 ± 1.9**	**47.4 ± 0.4**	**46.5 ± 0.6 ^(n)^**	**49.1 ± 0.4 ^(m)^**				
18:2n-6c	16.2 ± 0.8	16.3 ± 1.2	16.5 ± 1.7	16.2 ± 0.7	11.9 ± 0.5	13.1 ± 0.6	11.7 ± 0.5	12.0 ± 0.7	***	**	***	***
18:3n-3	5.9 ± 0.5	5.3 ± 0.5	5.5 ± 0.9	5.5 ± 0.5	4.2 ± 0.3	4.3 ± 0.4	4.0 ± 0.2	3.9 ± 0.5	**		**	**
18:3n-6	2.2 ± 0.2 ^b^	3.7 ± 0.9 ^a^	3.0 ± 0.6	3.3 ± 0.7	1.9 ± 0.1 ^b^	3.5 ± 0.6 ^a^	1.9 ± 0.3	2.9 ± 1.1				
20:5n-3 (EPA) ^3^	2.0 ± 0.1 ^B^	1.7 ± 0.4 ^M^	2.5 ± 0.2 ^A,m^	1.1 ± 0.2 ^N,n^	1.8 ± 0.1 ^a^	1.1 ± 0.1 ^b^	1.8 ± 0.1 ^m^	0.9 ± 0.1 ^n^		*	**	
22:5n-3	0.7 ± 0.0	0.5 ± 0.1	0.7 ± 0.1 ^m^	0.4 ± 0.1 ^n^	0.6 ± 0.1 ^a^	0.3 ± 0.0 ^b^	0.6 ± 0.0 ^m^	0.3 ± 0.1 ^n^		**		
22:6n-3 (DHA) ^4^	3.6 ± 0.3 ^B^	3.2 ± 0.8 ^M^	4.6 ± 0.4 ^A,m^	2.1 ± 0.5 ^N,n^	3.2 ± 0.2 ^a^	2.1 ± 0.3 ^b^	3.2 ± 0.2 ^m^	1.9 ± 0.2 ^n^		*	**	
**Σ PUFA ^5^**	**30.5 ± 1.6**	**30.7 ± 1.4**	**32.9 ± 3.6 ^m^**	**28.6 ± 0.7 ^n^**	**23.6 ± 1.1**	**24.4 ± 1.1**	**23.2 ± 0.3**	**21.9 ± 1.0**	***	***	***	***
EPA + DHA	5.6 ± 0.4 ^B^	4.9 ± 1.2 ^M^	7.1 ± 0.6 ^A,m^	3.2 ± 0.7 ^N,n^	5.0 ± 0.3 ^a^	3.2 ± 0.5 ^b^	5.0 ± 0.3 ^m^	2.8 ± 0.3 ^n^		*	**	
EPA/DHA	0.6 ± 0.0	0.5 ± 0.0	0.6 ± 0.0	0.5 ± 0.0	0.6 ± 0.0	0.5 ± 0.0	0.6 ± 0.0	0.5 ± 0.0				**
n-3/n-6	0.7 ± 0.0 ^a^	0.5 ± 0.1 ^b^	0.7 ± 0.0 ^m^	0.5 ± 0.0 ^n^	0.7 ± 0.0 ^a^	0.5 ± 0.0 ^b^	0.7 ± 0.0 ^m^	0.5 ± 0.1 ^n^				

^1^ Σ SFA is the sum of saturated fatty acids; ^2^ Σ MUFA is the sum of monounsaturated fatty acids; ^3^ EPA: Eicosapentaenoic acid; ^4^ DHA: Docosahexaenoic acid; ^5^ Σ PUFA is the sum of n-3 and n-6 polyunsaturated fatty acids. The values (mean ± SD, n = 3) with different superscript letters and different types of letters within one temperature treatment differ with *p*-values < 0.05 based on ANOVA, as described in the Materials and Methods section. The superscript letters indicate the output of tests based on comparisons of fish oil level within one supplement group (a, b: F6 vs. F2; m, n: F6 + RV vs. F2 + RV) and the effect of supplementation within one fish oil level (A, B: F6 vs. F6 + RV; M, N: F2 vs. F2 + RV), separated by temperature. The statistical outputs of the test based on the effect of the different holding temperatures analyzed within one feeding group are indicated in separate columns using * for *p* < 0.05, ** for *p* < 0.01 and *** for *p* < 0.001. All designations in brackets indicate a tendency towards a difference based on *p* < 0.1.

**Table 6:** The fatty acid composition (percentage of total fatty acid methyl esters (% FAMEs)) of fillet tissue of gilthead sea bream at the end of the 8-week feeding trial held at two different temperatures (19 °C and 23 °C). F6 and F2 indicate the feeding with the basal diets containing 6% and 2% dry matter (DM) fish oil, respectively. +RV indicates the feeding diets supplemented with 0.15% DM resveratrol.

% FAMEs	19 °C				23 °C				Comparison between 19 °C and 23 °C			
	F6	F2	F6 + RV	F2 + RV	F6	F2	F6 + RV	F2 + RV	F6	F2	F6 + RV	F2 + RV
14:0	2.5 ± 0.2 ^a^	2.2 ± 0.2 ^b^	2.7 ± 0.1 ^m^	2.1 ± 0.1 ^n^	2.6 ± 0.1 ^a^	2.1 ± 0.0 ^b^	2.4 ± 0.2 ^m^	2.0 ± 0.2 ^n^				
16:0	17.9 ± 0.1 ^a^	17.2 ± 0.2 ^b^	17.5 ± 0.1	17.1 ± 0.2	19.2 ± 0.3	19.5 ± 0.3	19.5 ± 0.4	19.2 ± 0.3	***	***	***	***
18:0	4.3 ± 0.2	4.4 ± 0.1	4.4 ± 0.0	4.4 ± 0.1	4.9 ± 0.2	4.9 ± 0.2	4.8 ± 0.2	4.9 ± 0.1	***	**	**	***
**Σ SFA ^1^**	**24.8 ± 0.2 ^a^**	**23.8 ± 0.3 ^b^**	**24.5 ± 0.2 ^m^**	**23.6 ± 0.2 ^n^**	**26.7 ± 0.3**	**26.5 ± 0.4**	**26.8 ± 0.5**	**26.2 ± 0.3**	***	***	***	***
14:1n-5	0.1 ± 0.0	0.1 ± 0.0	0.1 ± 0.0	0.1 ± 0.0	0.1 ± 0.0	0.1 ± 0.0	0.1 ± 0.0	0.1 ± 0.0				
16:1n-7	4.0 ± 0.3 ^a^	3.6 ± 0.3 ^b^	4.0 ± 0.1 ^m^	3.4 ± 0.1 ^n^	4.2 ± 0.1 ^a^	3.6 ± 0.1 ^b^	4.1 ± 0.1 ^m^	3.5 ± 0.2 ^n^				
18:1n-7c	2.9 ± 0.0	2.8 ± 0.1	3.0 ± 0.1 ^m^	2.8 ± 0.0 ^n^	2.7 ± 0.1 ^a^	2.4 ± 0.1 ^b^	2.7 ± 0.0 ^m^	2.5 ± 0.0 ^n^	***	***	***	***
18:1n-9c	31.2 ± 0.5 ^b^	33.0 ± 0.3 ^a^	30.3 ± 0.9 ^n^	32.6 ± 0.7 ^m^	31.1 ± 0.2 ^b^	34.0 ± 0.7 ^a^	31.2 ± 0.4 ^n^	33.8 ± 1.0 ^m^				
**Σ MUFA ^2^**	**38.2 ± 0.1**	**39.4 ± 0.3**	**37.4 ± 0.9 ^(n)^**	**38.8 ± 0.6 ^(m)^**	**38.0 ± 0.2 ^b^**	**40.0 ± 0.7 ^a^**	**38.1 ± 0.4 ^n^**	**39.8 ± 1.2 ^m^**				
18:2n-6c	19.7 ± 0.2	20.1 ± 0.5	19.7 ± 0.2 ^n^	20.6 ± 0.2 ^m^	18.5 ± 0.1	19.1 ± 0.2	18.5 ± 0.4 ^n^	19.4 ± 0.5 ^m^	**	**	**	**
18:3n-3	8.3 ± 0.1	8.3 ± 0.2	8.2 ± 0.2	8.4 ± 0.2	7.8 ± 0.1	8.2 ± 0.2	8.0 ± 0.3	8.2 ± 0.2	**			
18:3n-6	0.8 ± 0.0	0.9 ± 0.1	0.9 ± 0.0	1.0 ± 0.1	0.7 ± 0.0	0.7 ± 0.1	0.6 ± 0.1 ^n^	0.8 ± 0.1 ^m^		***	***	***
20:5n-3 (EPA) ^3^	2.8 ± 0.1	2.5 ± 0.2	3.2 ± 0.3 ^m^	2.5 ± 0.2 ^n^	2.7 ± 0.0 ^a^	1.8 ± 0.3 ^b^	2.6 ± 0.0 ^m^	1.9 ± 0.2 ^n^		**	*	**
22:5n-3	1.2 ± 0.0 ^(B)^	1.1 ± 0.1	1.4 ± 0.1 ^(A),m^	1.1 ± 0.1 ^n^	1.2 ± 0.1 ^a^	0.8 ± 0.1 ^b^	1.1 ± 0.0 ^m^	0.8 ± 0.1 ^n^		**	**	***
22:6n-3 (DHA) ^4^	4.3 ± 0.1	3.9 ± 0.3	4.8 ± 0.5 ^m^	4.0 ± 0.3 ^n^	4.4 ± 0.0 ^a^	3.0 ± 0.4 ^b^	4.3 ± 0.2 ^m^	3.1 ± 0.6 ^n^		*		*
**Σ PUFA ^5^**	**37.0 ± 0.2**	**36.8 ± 0.5**	**38.1 ± 0.7**	**37.7 ± 0.6**	**35.3 ± 0.3 ^a^**	**33.5 ± 0.9 ^b^**	**35.2 ± 0.7**	**34.0 ± 1.4**	*	***	***	***
EPA + DHA	7.1 ± 0.2	6.3 ± 0.4	8.0 ± 0.7 ^m^	6.6 ± 0.5 ^n^	7.1 ± 0.1 ^a^	4.8 ± 0.7 ^b^	7.0 ± 0.2 ^m^	4.9 ± 0.8 ^n^		**		**
EPA/DHA	0.7 ± 0.0	0.6 ± 0.0	0.7 ± 0.0	0.6 ± 0.0	0.6 ± 0.0	0.6 ± 0.0	0.6 ± 0.0	0.6 ± 0.0				
n-3/n-6	0.8 ± 0.0	0.8 ± 0.0	0.9 ± 0.0 ^m^	0.7 ± 0.0 ^n^	0.8 ± 0.0	0.7 ± 0.0	0.8 ± 0.0	0.7 ± 0.0				

^1^ Σ SFA is the sum of the saturated fatty acids; ^2^ Σ MUFA is the sum of the monounsaturated fatty acids; ^3^ EPA: Eicosapentaenoic acid; ^4^ DHA: Docosahexaenoic acid; ^5^ Σ PUFA is the sum of n-3 and n-6 polyunsaturated fatty acids. The values (mean ± SD, n = 3) with different superscript letters and different types of letters within one temperature treatment differ with *p*-values < 0.05 based on ANOVA, as described in the Materials and Methods section. The superscript letters indicate the output of tests based on comparisons of fish oil level within one supplement group (a, b: F6 vs. F2; m, n: F6 + RV vs. F2 + RV) and the effect of supplementation within one fish oil level (A, B: F6 vs. F6 + RV), separated by temperature. The statistical outputs of the test based on the effect of the different holding temperatures analyzed within one feeding group are indicated in separate columns using * for *p* < 0.05, ** for *p* < 0.01 and *** for *p* < 0.001. All designations in brackets indicate a tendency towards a difference based on *p* < 0.1.

**Table 7:** The ingredients and nutrient composition (percentage of dry matter: % DM) of the experimental diets. F6 and F2 are the basal diets with 6% and 2% DM fish oil, respectively. +RV indicates the supplementation of the basal diets with 0.15% DM resveratrol.

Ingredients (% DM)	F6	F2	F6 + RV	F2 + RV
Fish meal (Clupea sp.) ^1^	5.00	5.00	5.00	5.00
Soybean concentrate (HP 300) ^2^	19.00	19.00	19.00	19.00
Blood meal ^3^	7.00	7.00	7.00	7.00
Corn gluten ^2^	18.00	18.00	18.00	18.00
Wheat gluten ^4^	12.50	12.50	12.50	12.50
Rapeseed expeller ^5^	8.00	8.00	8.00	8.00
Wheat starch ^4^	10.96	10.96	10.96	10.96
Fish oil ^1^	6.00	2.00	6.00	2.00
Linseed oil ^6^	2.00	2.08	2.00	2.08
Rapeseed oil ^7^	0.10	1.65	0.10	1.65
Palm oil ^8^	0.90	3.27	0.90	3.27
Vitamin Mineral premix ^9^	0.50	0.50	0.50	0.50
Methionine ^9^	0.85	0.85	0.85	0.85
Lysine ^10^	1.19	1.19	1.19	1.19
Di-calcium phosphate ^11^	2.08	2.08	2.08	2.08
Inositol ^12^	0.02	0.02	0.02	0.02
Choline chloride ^13^	0.13	0.13	0.13	0.13
Cholesterol ^12^	0.11	0.11	0.11	0.11
Lecithin ^12^	2.53	2.53	2.53	2.53
α-Cellulose ^14^	2.00	2.00	2.00	2.00
Inert filler ^15^	1.13	1.13	1.13	1.13
Resveratrol (RV) ^16^			0.15	0.15
Nutrient composition (% DM)				
Dry matter	90.7	90.9	90.8	91.1
Crude ash	7.1	6.9	7.1	6.9
Crude protein	49.3	49.7	49.7	49.5
Crude lipid	14.5	14.6	14.3	14.8
Total carbohydrates	29.1	28.8	28.9	28.8
Gross energy (MJ kg^−^^1^ DM)	22.89	22.83	22.90	22.91

^1^ Vereinigte Fischmehlwerke Cuxhaven GmbH & Co. KG, Cuxhaven, Germany; ^2^ EURODUNA Rohstoffe GmbH, Barmstedt, Germany; ^3^ Sonac, Son, Netherlands; ^4^ KRÖNER STÄRKE GmbH, Ibbenbüren, Germany; ^5^ Stöfen Landhandel, Wesselburen, Germany; ^6^ Makana Produktion und Vertrieb GmbH, Offenbach a.d.Queich, Germany; ^7^ Different food stores, Büsum, Germany; ^8^ EFG Elbe Fetthandel GmbH, Geesthacht, Germany; ^9^ Emsland-Aller Aqua GmbH, Golßen, Germany; ^10^ Biolys: Evonik Industries AG, Essen, Germany; ^11^ J. Rettenmaier und Söhne GmbH und Co. KG, Rosenberg, Germany; ^12^ Roth GmbH, Karlsruhe, Germany; ^13^ Sigma Aldrich, St. Louis, USA; ^14^ Mikro-Technik GmbH & Co. KG, Bürgstadt/Main, Germany; ^15^ Bentonite: Del Lago Bentonite, Castiglioni Pes y Cía., Buenos Aires, Argentina; ^16^ CHEMOS GmbH & Co. KG, Regenstauf, Germany.

**Table 8:** The primer sequences for hepatic mRNA measurements via quantitative real-time RT-PCR (qRT-PCR). Forward and reverse primers, as well as their specific annealing temperatures, were used for qRT-PCR measurements of mRNA levels in the total RNA samples extracted from gilthead sea bream liver.

Primer	Sequence 5′ → 3′	Annealing Temperature (°C)
∆6-D ^a^* forward	GCAGGCGGAGAGCGACGGTCTGTTCC	65
∆6-D ^a^* reverse	AGCAGGATGTGACCCAGGTGGAGGCAGAAG	65
β-actin ^b^* forward	TCCTGCGGAATCCATGAGA	60
β-actin ^b^* reverse	GACGTCGCACTTCATGATGCT	60
PPARα ^c^* forward	TCTCTTCAGCCCACCATCCC	61
PPARα ^c^* reverse	ATCCCAGCGTGTCGTCTCC	61
ECH ^d§^ forward	GCCCAAGAAGCCAAGCAATCAG	60
ECH ^d§^ reverse	CTTTAGCCATAGCAGAGACCAGTTTG	60
CEL ^e§^ forward	GCTGAGGAGATTGCTCTGAAGGT	62
CEL ^e§^ reverse	CAGGAAGCCATAGTCTCACCAGTG	62

^a^ ∆6-D: ∆6-desaturase; ^b^ β-actin: Beta-actin; ^c^ PPARα: Peroxisome proliferator-activated receptor α; ^d^ ECH: Enoyl-CoA hydratase; ^e^ CEL: Carboxyl ester lipase; * [11]; ^§^ [56].
